# Apraxia phenotypes and frontotemporal lobar degeneration

**DOI:** 10.1007/s00415-024-12706-5

**Published:** 2024-10-10

**Authors:** Tobias C. Langheinrich, Jennifer C. Thompson, Matthew Jones, Anna M. T. Richardson, David M. A. Mann, Julie S. Snowden

**Affiliations:** 1 Cerebral Function Unit, Manchester Centre for Clinical Neurosciences, Northern Care AllianceNHS Foundation Trust, Salford, UK; 2 Division of Psychology, Communication & Human Neuroscience, Faculty of Biology, Medicine and Health, University of Manchester, Manchester, UK; 3 Division of Medical Education, Faculty of Biology, Medicine and Health, University of Manchester, Manchester, UK; 4 Division of Neuroscience, Faculty of Biology, Medicine and Health, University of Manchester, Manchester, UK

**Keywords:** Apraxia, Progressive aphasia, Corticobasal syndrome, Tau pathology, TDP pathology, Progranulin

## Abstract

**Background:**

Apraxia has been identified in all clinical forms of frontotemporal lobar degeneration (FTLD). The characteristics of apraxia symptoms and their underlying cognitive/motor basis are not fully understood. This study investigated apraxia in pathological subtypes of FTLD.

**Methods:**

The study constituted a retrospective review of 115 pathologically confirmed cases of FTLD from a single cognitive neurology centre. Patients in whom apraxia had been documented as a notable clinical characteristic were identified. Apraxia features, demographic, cognitive, neurological, and imaging findings were recorded.

**Results:**

Eighteen patients were identified: 12 with FTLD-tau pathology (7 corticobasal degeneration (CBD), four Pick type and one progressive supranuclear palsy (PSP)) and six with FTLD-TDP pathology, all type A and four linked to progranulin gene mutations. Apraxia as a dominant presenting feature was typically associated with tau pathologies, whereas it emerged in the context of aphasia in TDP pathology. Apraxia typically predominated in one body part (face or limb) in tau but not TDP pathology. Relatively preserved activities in daily life were associated with TDP. Apraxia of speech was associated with tau pathology. Pick-type pathology was linked to symmetrical atrophy and late development of limb rigidity.

**Conclusion:**

Apraxia in FTLD subtypes has variable characteristics. Apraxia associated with CBD pathology conformed to criteria for probable corticobasal syndrome (CBS), whereas apraxia with Pick-type pathology did not. Apraxia in patients with TDP-A pathology was interpreted as one manifestation of their generalised communication disorder. Apraxia in FTLD may have distinct cognitive and motor substrates that require prospective investigation.

## Introduction

An association between apraxia and frontotemporal lobar degeneration (FTLD) pathology is well recognised. The prototypical apraxic syndrome, corticobasal syndrome (CBS), is characterised by orobuccal and/or limb apraxia, often with alien limb phenomena, occurring in the context of asymmetric limb rigidity, dystonia and/or myoclonus [1]. The underlying pathology, according to current classification [2–4], is FTLD-tau of corticobasal degeneration (CBD) type [5]. Nevertheless, apraxia—or a CBS-like syndrome—is not confined to CBD pathology. It may occur too in association with other pathological subtypes of FTLD, both tau and TDP-43 [6–9], as well as in other conditions such as Alzheimer’s disease. Moreover, apraxia has been reported in association with each of the canonical forms of frontotemporal dementia (FTD): behavioural variant, non-fluent aphasia, and semantic dementia/aphasia as well as with genetic mutations in familial FTD [10, 11].

Traditional classifications of apraxia distinguish limb-kinetic, ideomotor and ideational forms [12, 13], ‘ideational’ referring to the conceptualisation of an action and ‘ideomotor’ to its implementation. Nevertheless, ideational and ideomotor deficits may co-occur [14], leading some authors to suggest that traditional classifications should be abandoned [15]. In the evaluation of apraxia task-based distinctions are commonly made, such as between production of transitive (object use) and intransitive (e.g. wave goodbye) actions [16], pantomime to verbal command and on imitation [17, 18], imitation of meaningful and meaningless gestures [18, 19] and production of single versus multi-step actions [19]. It is acknowledged that praxis involves a distributed brain network involving both dorsal and ventral processing streams [18, 20–22], reflecting its multifaceted nature. Nevertheless, different tasks have different processing requirements and so brain regions may be affected differentially. Tool-related action pantomimes, which make semantic demands, have been found to be crucially dependent on temporal lobe structures, whereas imitation of meaningless hand postures depends heavily on integrity of the parietal region [18, 23]. The kinematic component of gesture tasks (amplitude and timing of movement), important for imitation of meaningless gestures, has been linked to inferior parietal and frontal regions, and the posture component (hand/arm positioning), crucial for pantomimed gesture of tool use, to posterior temporal lobe [18]. Transitive actions (imitation of tool use) and intransitive gestures, such as waving goodbye, have been found to rely differentially on ventral–dorsal and ventral pathways, ascribed to the higher demands on temporal–spatial processing in imitation of tools [22].

Distinct profiles of apraxia have been reported, reflecting different task demands. A study of CBS [24] found transitive gestures to be more affected than intransitive and imitation more than pantomime, suggesting deficits in visuomotor transformation. Imitation of non-representational hand postures were particularly impaired. The only notable difference between patients with left and right hemisphere presentations of CBS was an adverse effect of verbal cueing on imitation performance only in the left hemisphere group. Another study [25] found that impairments in presumptive CBD were not confined to production tasks. Deficits were also detected in matching gestures to objects and in the selection of the appropriate tool for use in a mechanical problem-solving task, suggesting a central problem in action knowledge. An earlier study by those authors [26] revealed distinct performance profiles between two patients with semantic dementia, who had temporal lobe atrophy, and one with presumptive CBD with parietal atrophy. The CBD patient showed poor novel tool selection in the problem-solving task and impaired use of real objects, despite well-preserved knowledge of the objects’ function. The patients with semantic dementia showed a contrasting picture of impaired object identification, including knowledge of the object’s function but preserved performance on the novel tool task. Object use has been argued to be heavily dependent on object-specific conceptual knowledge [27]. A comparative study of presumptive CBD and PSP [28] showed not only more frequent apraxia in CBD than PSP but also different qualitative characteristics of performance. Whereas in CBD, movements were clumsy, and in PSP, there were more sequencing errors and perseverations.

A complicating factor in characterising apraxia is that there is no one-to-one correspondence between praxis task and underlying process [20, 21]. Indeed, task failures may arise secondary to other cognitive deficits [29, 30]. For example, tool use disorders in semantic dementia/svPPA have been attributed directly to patients’ semantic loss [31, 32]. Task-based approaches have clinical value in the assessment of praxis, but there is a need for care in their interpretation.

Characterisation of apraxia in pathological forms of FTLD is limited, so the degree of variation in clinical presentation is unclear. The study aimed to determine whether apraxias associated with FTLD pathology are uniform or have distinct characteristics.

## Materials and methods

### Study type

This is a retrospective cohort study of patients with apraxia associated with FTLD pathology.

### Case ascertainment

The research database was examined for patients who attended the cerebral function unit, a specialist unit for early onset and atypical dementias within the Manchester Centre for Clinical Neurosciences. Patients were identified who had donated their brain post-mortem to the Manchester Brain Bank between 1990 and 2016 and were selected according to the following criteria: (i) apraxia was a documented prominent characteristic of their clinical disorder at some time during the disease course and (ii) the pathological substrate was consistent with a form of FTLD [2, 3]. The selection process did not encompass subtle failures of performance on tests of praxis, such as problems on manual sequencing tasks or problems arising secondary to poor comprehension. Patients were excluded if neuropathological findings were equivocal. Patients included in the study had been assessed, following referral, by a neurologist and neuropsychologist. A cognitive, behavioural, and neurological clinical history with both patient and informant had been carried out in all cases using a structured clinical history proforma that addresses distinct domains including praxis. A full neurological examination included an evaluation of praxis. Neuropsychological examination included use of the Manchester neuropsychological profile [33], which complements the clinical history in addressing individual cognitive domains systematically. Praxis tasks include orofacial and limb symbolic gestures and action pantomimes, executed to verbal command and on imitation, as well as copying of non-representational hand postures. Limb gestures, upper and lower are assessed separately for right and left limb. Patients were excluded if details of praxis performance in case notes were insufficient to be clinically informative. Patients had typically undergone magnetic resonance and/or single photon emission tomographic imaging, which provided support for the diagnosis, which was made on clinical grounds.

Patients had been followed up during life, although the number of follow-up reviews varied across the cohort. The clinical diagnoses reported here represent those made following patients’ initial assessment. Amendments to diagnosis over the disease course are indicated in the text.

### Classification of patients

#### Clinical

Patients’ original clinical diagnoses were recorded. Patients were also classified according to contemporary classifications, given that many diagnoses preceded publication of contemporary diagnostic designations, e.g., CBS.

#### Pathological

Pathological classifications are based on consensus criteria for FTLD [3, 4, 34] that distinguish between tau and non-tau forms, and subtypes of those pathologies. Subtyping for FTLD-tau pathologies includes Pick’s, corticobasal degeneration (CBD), and progressive supranuclear palsy (PSP) subtypes, and for FTLD-TDP pathologies type A-D. Pathological classifications had been made by a neuropathologist (DM) independently of the clinical classifications.

### Recording of clinical features

The following features were recorded from patients’ clinical records:

#### Demographics

Sex, handedness, age at onset of symptoms, duration at the time of diagnostic assessment and death, family history of dementia, and genetic findings where available.

#### Praxis

- The timing of the emergence of apraxia: whether it was an early feature (a presenting complaint and/or demonstrated at the time of referral), or emerged only later in the disease course,
- The presence of apraxia for orofacial, upper and lower limb movements,
- The body part initially or predominantly affected (orofacial or limb),
- For orofacial movements, the presence of apraxia for repetitive and alternating speech sounds, facial actions (eye closure, raising eyebrows, protruding tongue), expressions of emotion (happy, surprise, anger) and action pantomimes (blow match, suck through straw, sniff flower),
- Asymmetry of limb apraxia,
- Symbolic/communicative (gestures and action pantomimes) versus non-symbolic/meaningless (copying of non-representational hand postures) actions,
- Actions to verbal command versus imitation,
- Transitive versus intransitive (pantomime object use versus symbolic gesture),
- Impact of apraxia on activities of daily living,
- Spontaneous use of gesture to compensate for spoken communication problems,
- Evidence of knowledge of action that cannot be executed (e.g., patient describes action verbally, reports being able to visualise action or comments appropriately on praxic errors; performs well on gesture matching test),
- Nature of errors of limb praxis: degraded/crude actions, incorrect positioning in space, impaired configuration of fingers, body part substitutions when demonstrating object use, perseveration of previous action, alien limb phenomena (involuntary levitation or wandering of limb, tonic grasping, inter-manual conflict, mirror movements and synkinesis).

#### Neurological signs

The following neurological signs were recorded: pyramidal and/or extrapyramidal signs, symmetrical or asymmetrical, eye movement disorder, tremor, myoclonus, stooped posture, abnormal gait, falls, and grasp reflexes.

#### Cognitive changes

Language, visuospatial functions, memory, and executive function were evaluated by the Manchester neuropsychological profile [33]. Assessments incorporated published tests, including the graded naming test [35], visual object and space perception battery [36], category (animals) and letter (FAS) fluency [37, 38] and Weigl’s block sorting test [39]. The presence of apraxia of speech, characterised by effortful, laboured speech production, was determined based on clinical reports and descriptions of patients’ speech production in conversational speech and series speech (recitation of months of the year).

#### Neuroimaging

Findings were coded with respect to right/left or anterior/posterior asymmetry of structural change (CT or MRI), hypoperfusion (HMPAO SPECT). The timing of imaging in relation to onset of symptoms was determined.

#### Longitudinal data

Longitudinal data were used to document the evolution of praxic, neurological and cognitive symptoms and signs. For simplicity, changes were operationally designated as ‘early’ if they were present within 3 years of onset of symptoms and ‘late’ if they emerged later in the disease course.

### Statistical analysis

The presence or absence of symptoms and signs in different subgroups was compared using 2-tailed Fisher’s exact tests. Effect size was determined using the phi (*Φ*) statistic. Interval data were compared using t-tests. Findings are reported uncorrected for multiple comparisons in view of the exploratory nature of the study.

## Results

From a pathological cohort of 307 patients, 115 were identified with FTLD pathologies. The breakdown of pathologies and associated clinical phenotype is shown in Table 1.

**Table 1:** FTLD pathologies and associated clinical diagnoses

FTLD pathology^a^		Clinical phenotype												
		bvFTD	FTD/MND	nfv PPA	nfvPPA/bvFTD	SD	bvFTD/SD	CBS	PAX	PSPs	PSPs/bvFTD	CBS/PSP	AD	Total
TDP-43	TDP-A	13	–	7	3	–	–	1	–	–	–	–	1	25
	TDP-B	6	14	–	–	–	2			1	–	–	–	23
	TDP-C	–	–	–	–	8	2	–	–	–	–	–	–	10
	Unclassified	–	–	–	–	1	–	–	–	–	–	–	–	1
Tau	Pick	8	–	2	2	–	–	1	2	–	–	–	–	15
	MAPT	5	–	–	–	–	8	–	–	–	–	–	–	13
	CBD	3	–	2	2	–		5	–	–	–	–	2	14
	PSP	1	–	–	1	–	–	–	–	4	1	1	1	9
	GGI	1	–	–	–	–	–	–	–	–	–	–	–	1
FUS	FUS	2	–	–	–	–	–	–	–	–	–	–	–	2
No inclusions	FTLD–ni	2	–	–	–	–	–	–	–	–	–	–	–	2
	Total	41	14	11	8	9	12	7	2	5	1	1	4	115

^a^pathological classification as per Mackenzie et al. [3, 4]

*nfvPPA* non-fluent variant PPA, *nfPPA/bvFTD* mixed phenotype nfvPPA and bvFTD, *SD* semantic dementia, bvFTD/SD mixed phenotype bvFTD and SD, *CBS* corticobasal syndrome, *PAX* progressive apraxia (assumed different from CBS), *PSPs* progressive supranuclear palsy syndrome, *CBS/PSP* mixed CBS and PSPs phenotype, *AD* Alzheimer’s disease, *–*  no recorded cases

From this pathological cohort, 22 patients were identified in whom apraxia was recorded as a notable clinical feature. Four patients were excluded because of incomplete clinical data. 18 patients fulfilled the inclusion criteria for the study.

### Clinical classification

Ten of the 18 patients had apraxia as the dominant presenting symptom (Table 2). In 7 of these 10, the original presumptive diagnosis was CBD. The contemporary designation of CBS is indicated in the table. Two patients (8 and 10), by virtue of unusual characteristics of their apraxia, had been classified as primary progressive apraxia (PAX) and thought to be distinct from CBD. One patient (12) had a mixed phenotype, with early asymmetrical rigidity and apraxia combined with a supranuclear gaze palsy and early falls, fulfilling both possible CBD and possible PSP-CBS clinical diagnostic criteria. The other eight patients presented initially with progressive language disorder, consistent with the broad designation PPA, apraxia being a secondary or later development. Those patients’ original clinical diagnosis was progressive non-fluent aphasia in keeping with current nomenclature of non-fluent variant (nfv) PPA. In only one patient was the clinical diagnosis amended on follow-up. Patient 10 had an initial diagnosis of PAX, amended to PAX/nfvPPA because of the later development of language problems. Apraxia remained the dominant feature throughout the disease course.

**Table 2:** Patient characteristics: clinical and pathological phenotype, demographics and imaging asymmetries

Case ID	Presentation	Clinical diagnosis	Pathological classification of FTLD^a^	Sex	Hand	Age at onset	Years of illness at test	Total years onset till death	FH Goldman score^b^	Genetic mutation	Imaging asymmetry	Imaging anterior predom	Imaging parietal atrophy/hypoperfusion	Pathology parietal atrophy present
1	Apx	CBS	Tau-CBD	M	R	51	8	9	4	no	R > L	Yes	Yes	Yes
2	Apx	CBS	Tau-CBD	F	R	77	2	7	4	no	L > R	No	Yes	?
3	Apx	CBS	Tau-CBD	F	R	47	5	10	4	no	L > R	Yes	Yes	Yes
4	Apx	CBS	Tau-CBD	M	R	66	3	5	4	no	R > L	No	Yes	Yes
5	Apx	CBS	Tau-CBD	F	R	65	4	6	4	no	R > L	No	Yes	?
6	PPA	nfvPPA	Tau-CBD	M	R	73	2	6	4	no	R > L	Yes	Yes	Yes
7	PPA	nfvPPA	Tau-CBD	M	R	74	2	5	4	n/a	L > R	Yes	?	Yes
8	Apx	PAX	Tau-Pick	M	R	55	5	11	3.5	no	L = R	Yes	Yes	Yes
9	Apx	CBS	Tau-Pick	F	R	72	4	8	4	n/a	L = R	No	Yes	Yes
10	Apx	PAX	Tau-Pick	F	R	53	2	4	4	no	L = R	Yes	Yes	Yes
11	PPA	nfvPPA	Tau-Pick	M	R	57	1	15	4	no	L > R	Yes	Yes	Yes
12	Apx	CBS/PSP	Tau-PSP	M	R	62	2	7	2	n/a	L = R	Yes	Yes	?
13	PPA	nfvPPA	TDP-A	M	R	62	2	8	3.5	n/a	L > R	Yes	Yes	Yes
14	PPA	nfvPPA	TDP-A	F	L	63	2	8	1	*GRN*	L > R	Yes	Yes	Yes
15	PPA	nfvPPA	TDP-A	M	R	66	3	8	1	*GRN*	L > R	Yes	Yes	Yes
16	PPA	nfvPPA	TDP-A	F	R	66	3	11	4	n/a	L > R	Yes	Yes	Yes
17	PPA	nfvPPA	TDP-A	M	R	62	2	5	2	*GRN*	L > R	Yes	Yes	Yes
18	Apx	CBS	TDP-A	M	R	66	3	5	2	*GRN*	R > L	No	Yes	Yes

^a^As per Mackenzie et al. [3, 4]

^b^Goldman score [40, 41]: 1 and 2 = three affected individuals affected over two generations; 3.5 = family history reported in family member with onset after 65 years; 4 = no known family history

*M* male, *F* female, *FH* family history, *n/a* no information available, *Hand* handedness, *R* right-handed, *L* left-handed, *Apx* apraxic syndrome, *PPA* primary progressive aphasia, *L* left, *R* right, *anterior predom* predominance of atrophy/hypoperfusion in anterior brain regions, *?* not reported.

### Pathological classification

The FTLD pathological substrate was heterogeneous (Table 2). Tau pathology was most common, and was of CBD type in seven patients, Pick type in four and PSP type in one. FTLD-TDP pathology was present in six cases, all type A (TDP-A). No patient with MAPT tau pathology, associated with mutations in the *MAPT* gene, nor patients with TDP-B and TDP-C pathology were included in the study. Table 2 shows the presence of parietal atrophy on pathological examination in the patients with apraxia. Co-incidental AD pathology of mild degree (Braak staging I/II) was reported in two patients with CBD and one with PSP pathology (5, 7 and 12). No patient with TDP-A pathology showed pathological changes of AD.

### Demographics, genetics, and imaging

Patient details are shown in Table 2. A strong family history of was present in 5 (28%) cases. Where a family history was reported only in one first-degree relative with onset over 65 years (Goldman rating 3.5) [40, 41], this was treated as absent for the purposes of analysis. Mutations in the *progranulin* (*GRN*) gene in patients 14, 15, 17 and 18 were identified in exon 4Q130SfsX124, exon 1C31LfsX34, exon 10V452WfsX38, and exon 10V452WfsX38, respectively.

Structural brain imaging (MR or CT) was carried out in 17/18 cases and functional imaging (SPECT) in 16/18. In all the patients, at least one form of imaging took place within 12 months of clinical assessment. However, in three patients (4, 5 and 6), structural imaging preceded clinical assessment by more than 12 months and in one patient (13), SPECT was carried out more than 12 months after clinical evaluation. Asymmetry, anterior emphasis and presence of parietal atrophy and/or hypoperfusion are shown in Table 2.

### Inter-relationships: clinical presentation, pathology, demographic features, and imaging asymmetry

The dominant clinical feature at presentation (apraxia or aphasia) was a significant predictor of underlying pathology. An apraxic presentation was more likely to be associated with tau pathology and aphasia with TDP pathology (2-tailed Fisher’s exact test *p* = 0.04, *Φ* = 0.55). An apraxic presentation was not, however, limited exclusively to tau pathology (Table 2).

Clinical presentation influenced the duration of symptoms at referral (t(df. = 11.5) = 2.64, *p* = 0.02): patients presenting with aphasia were referred earlier. Clinical presentation was not affected by sex (Fisher’s exact *p* = 0.37), nor did it influence age at onset of symptoms: (t(df = 16) = −1.03, *p* = 0.32) and total illness duration till death (t(df. = 16) = −0.79, *p* = 0.44).

Sex, age at onset, years of illness and total duration of illness did not differ significantly in patients with tau and TDP pathologies. The presence of a strong family history was, however, more commonly associated with TDP pathology (Fisher’s exact test *p* = 0.02, *Φ* = 0.61) and linked to the *GRN* gene. A positive family history was reported in no patient with CBD pathology.

The presence of lateral asymmetry on neuroimaging did not distinguish an apraxic versus PPA clinical presentation (Fisher’s exact *p* = 0.31), nor tau versus TDP pathology (Fisher’s exact *p* = 0.25, *Φ* = 0.38). By contrast, it did distinguish within tau pathologies. Patients with CBD pathology were more likely to show imaging asymmetry than those with non-CBD pathologies: CBD versus Pick + PSP, Fisher’s exact test *p* = 0.01, *Φ* = 0.84; CBD versus Pick, Fisher’s exact *p* = 0.02, *Φ* = 0.81.

### Apraxia overview—body parts affected

Apraxia occurred in all patients for both orofacial and limb movements (Table 3). Notably, however, patients with tau pathology (CBD, Pick, PSP) typically presented with apraxia predominantly affecting one body part (orofacial or limb), whereas in patients with TDP pathology, the apraxia, when it emerged, affected orofacial and limb movements similarly (Fisher’s exact *p* = 0.002, *Φ* = 0.79). Orofacial apraxia was the dominant apraxic characteristic in 5/12 patients with tau pathology, in three of whom apraxia of speech was reported as the presenting characteristic.

**Table 3:** Overview of apraxia and its evolution

ID	Clinical diagnosis	Path	Dominant presenting	Present early (< 3 years post-onset)			Dominant late	Present late (> 3 years post-onset)			Asymmetry of limb apraxia
			orofacial vs limb	OF	UL	LL	Orofacial vs limb	OF	UL	LL	
1	CBS	CBD	Limb	−	+	−	Both	+	+	+	L > R
2	CBS	CBD	Orofacial	+	+	+	Both	+	+	+	L > R
3	CBS	CBD	Orofacial	+	+	−	Both	+	+	+	R > L
4	CBS	CBD	Limb	+	+	+	Limb	+	+	+	L > R
5	CBS	CBD	Limb	+	+	+	Limb	+	+	+	L > R
6	nfvPPA	CBD	Orofacial	+	−	−	Orofacial	+	+	−	L = R
7	nfvPPA	CBD	Orofacial	+	+	+	Orofacial	+	+	+	L = R
8	PAX	Pick	Limb	−	+	−	Limb	+	+	+	L = R
9	CBS	Pick	Limb	−	+	−	Both	+	+	+	R > L
10	PAX	Pick	Orofacial	+	−	−	Orofacial	+	+	+	L = R
11	nfvPPA	Pick	N/a	−	−	−	Both	+	+	−	L = R
12	CBS/PSP	PSP	Both	+	+	−	Both	+	+	−	L > R
13	nfvPPA	TDP-A	N/a	−	−	−	Both	+	+	?	R > L
14	nfvPPA	TDP-A	N/a	−	−	−	Both	+	+	+	R > L
15	nfvPPA	TDP-A	Both	+	+	+	Both	+	+	+	R > L
16	nfvPPA	TDP-A	N/a	−	−	−	Both	+	+	+	L = R
17	nfvPPA	TDP-A	Both	+	+	+	Both	+	+	+	L = R
18	CBS	TDP-A	Both	+	+	+	Both	+	+	+	L > R

Apraxic presentations unshaded; PPA presentations shaded

Both = orofacial and limb apraxia equal; n/a not applicable (no apraxia)

+  = present; −= absent; ? = information not available

Note that although orofacial and limb apraxia may both be present, one or other may predominate in severity in early and/or late disease

*OF* orofacial, *UL* upper limb, *LL* lower limb, *L* left, *R* right

Left-predominant limb apraxia was more commonly associated with an apraxic than PPA presentation (Fisher’s exact *p* = 0.01, *Φ* = 0.73). PPA clinical presentations were associated with symmetrical apraxia or more severe apraxia in the right limb.

### Characteristics of apraxia

#### Orofacial

When orofacial apraxia was present, either early or late in the disease course, it encompassed vocalisations (alternating speech sounds and cough), facial actions (eye closure, raising eyebrows, and protruding tongue), expressions of emotion (happy, surprise, and anger) and action pantomimes (blow match, suck through straw, sniff flower). No significant dissociations were elicited with respect to action type or subgroups (Table 4). In only a third of patients, however, did the quality of speech output in conversational speech conform to conventional definitions of speech apraxia i.e. effortful, laboured production with articulatory groping and sound distortions (see “Language”).

**Table 4:** Apraxia characteristics

ID	Clinical diagnosis	Path	Orofacial task—impairment				Limb—action disparities					Limb—error types					
			Vocal	OF actions	Emot demo	Pant demo	Non-sym sup	Imit sup	Tran-intran disp	ADL sup	Spont gest	Degr	Spat	Int con	BPS	Pers	Alien limb
1	CBS	CBD		+	+	+	−		−	−		+	−	+	−	−	−
2	CBS	CBD	+	+	+	+	−		−	−	−	+	−	−	+	−	−
3	CBS	CBD	+	+	+	+	−	−	−	−	−	+	−	−	+	−	+
4	CBS	CBD		+	+		−		−	−		+	−	+	−	+	+
5	CBS	CBD	−	+	+	−	−	−	−	−	−	+	+	+	+	−	+
6	nfvPPA	CBD	+	−	+	+		−	−	−		−	−	−	+	−	−
7	nfvPPA	CBD	+	+	+	+		−			+	−	−	−	+	+	−
8	PAX	Pick		+		+	−		−	−		+	−	−	+	−	+
9	CBS	Pick	−	+	+	+	−	−	−	−	+	+	+	−	+	−	+
10	PAX	Pick	+	+	+	+			−	−	+	+	−	−	+	+	−
11	nfvPPA	Pick	+	+	+	+	+	+	−	+	+	−	−	−	+	−	−
12	CBS/PSP	PSP	+	+	+	+	+	−	−			+	+	−	−	+	−
13	nfvPPA	TDP-A				+			−	+		−	−	−	−	+	−
14	nfvPPA	TDP-A	nr	nr	nr	nr		+	−	+	−	+	−	−	−	+	−
15	nfvPPA	TDP-A		+		+	+	+	−	+	−	+	−	−	+	+	−
16	nfvPPA	TDP-A	+	+	+	+	+	+	−	+		+	+	−	−	−	−
17	nfvPPA	TDP-A	+	+	+	+	−	+	−	+	−	+	+	+	+	+	−
18	CBS	TDP-A	+	+	+	+	−	−	−		−	+	+	+	+	+	+

Apraxic presentation unshaded; PPA presentation shaded

*Vocal* actions requiring vocalisation (alternating speech sounds; cough), *OF actions* orofacial actions to command (close eyes, raise eyebrows, protrude tongue, waggle tongue), *Emot demo* facial expressions of emotion to command (happy, surprise, anger), *Pant demo* pantomime transitive actions (blowing match, sucking through straw, sniffing flower), *Non-sym sup* non-symbolic actions (copying nonrepresentional/meaningless hand postures) superior to symbolic actions (copying gestures and action pantomimes), *Imit sup* actions from imitation superior to verbal command, *Tran-intran disp* disparity in performance for transitive (pantomime object use) compared to intransitive (gesture) actions, *ADL sup* daily living actions (e.g. dressing, drinking from cup) relatively preserved compared to gestures/action pantomimes, *Spont gest* spontaneous use of gesture to compensate for problems in communication, *Degr* degraded, clumsy actions, *Spat* impaired positioning of limb in space or with reference to body, *Int con* impaired spatial configuration of fingers, *BPS* body part substitution when pantomiming object use, *Pers* perseveration of previous action

+  = impairment/action disparity/error type present; − = impairment/action disparity/error type absent; nr = no response elicited late in course; empty cells = information not available

#### Limb

For limb movements, superior performance for non-symbolic compared to symbolic actions was reported more often in association with PPA than apraxic presentations (Fisher’s exact test *p* = 0.05, *Φ* = 0.64) (Table 4). Similarly, patients with PPA were more likely to show improved performance on imitation (Fisher’s exact test *p* = 0.03, *Φ* = 0.71). No differences were observed with respect to transitive versus intransitive actions.

More striking differences were observed in relation to activities of daily living. Relative preservation of activities of daily living, in contrast to the severe apraxia demonstrated on testing, was recorded more often in association with PPA than apraxia clinical presentation (Fisher’s exact test *p* = 0.003, *Φ* = 0.86) and more often with TDP than tau pathology (Fisher’s exact test, *p* = 0.007, *Φ* = 0.84). No differences between transitive (pantomime) and non-transitive (gesture) actions were reported.

Some patients with tau pathology were reported to use limb gesture spontaneously as a means of compensating for communication difficulties, particularly patients 7 and 10, in whom orofacial apraxia was a prominent presenting feature. Compensatory gesturing was not reported in patients with TDP pathology.

Reporting of patients’ level of awareness of deficit was limited. Nevertheless, patients 4 and 6 with CBD and 16 with TDP-A pathology were reported to show frustration at their limb apraxia. Patients 8 and 9 with Pick pathology described verbally actions that they could not complete.

In virtually all patients, actions were described in clinical reports as ‘degraded’ or ‘clumsy’ (Table 4). Reports of spatial impairment, either in terms of internal configuration of the fingers or positioning in space, were recorded in some patients but without predictive value. Body part substitution, when demonstrating object use, was common, but again was poorly predictive. Perseveration of actions was reported in 4/12 (33%) of patients with tau pathologies and 5/6 (83%) of patients with TDP pathology (Table 4). This difference did not reach statistical significance.

Alien limb phenomena were reported in six patients, exclusively in those with an apraxic presentation. The comparison between frequency of alien limb in PPA and apraxic presentations was significant (Fisher’s exact test *p* = 0,011, *Φ* = 0.63). Of the six patients, the underlying pathology was tau-CBD in three, tau-Pick in two and TDP-type A in one. Specific characteristics varied. Patients with CBD and patient 8 with Pick pathology exhibited involuntary wandering of the limb in space, together with synkinesis or mirroring of movements by the opposite hand. Striking early features of patient 8 with Pick pathology were inter-manual conflict, involuntary grasping and difficulty releasing his grip (tonic grasping), with environmental interference. For example, when intending to put an object in a cupboard he might hold the object in one hand whilst placing the empty hand in the cupboard. When opening the door for his wife he might grab her wrist rather than the door handle. He was acutely aware of errors, and he could provide an accurate verbal account of the correct actions and positioning of his limbs. Patient 18 with TDP-A pathology described his hand as ‘him’. He showed both tonic grasping of external objects, and involuntary releasing of objects held in his hand.

### Neurological signs

All patients developed neurological signs during their illness. Nevertheless, they were more often an early feature of patients with tau than TDP pathology, particularly in patients with CBD pathology (Table 5). Limb rigidity was present early in 7/7 patients with tau-CBD pathology, but only 1/4 patients with tau-Pick pathology (Fisher’s exact, *p* = 0.02, *Φ* = 0.81) and 1/6 patients with TDP-A pathology (Fisher’s exact, *p* = 0.005, *Φ* = 0.85). No patient showed limb weakness during their disease course. Falls were an early feature of patient (12) with mixed apraxia and PSPs clinical phenotype and PSP pathology, and one other patient only (5), with apraxia presentation and CBD pathology in whom they occurred as a late feature.

**Table 5:** Neurological signs

ID	Pathology	SNGP	Eye apra	Hypomim	Dysphag	Hypophon	Slow tong	App rigid	Spasticity	Tremor	Myoclonus	Grasp reflex	Brisk reflex	Plant extens	Vis inatt	Stoop pos	Park gait	Falls
1	CBD	L	L	**E**			–	**E**	**E**	**E**	L	L	**E**	–			L	
2	CBD	**E**	**E**	**E**		**E**	**E**	**E**		**E**		L	L	L		L	L	
3	CBD	L	L	L	L	L		**E**	L		L	L	L	L		L	L	L
4	CBD	**E**		**E**				**E**			**E**	**E**	**E**	–	**E**		**E**	
5	CBD	**E**	**E**	**E**	L		**E**	**E**	L	–	–		**E**	**E**			**E**	**E**
6	CBD	–		**E**				**E**		**E**			–	–		–	**E**	
7	CBD							**E**				**E**	–	**E**				
8	Pick	L	L	L				L			L		L	–		L	L	L
9	Pick	L			L		**E**	**E**	L		**E**		**E**	**E**	**E**	**E**		L
10	Pick				**E**	**E**		L	–				L	–				
11	Pick			L	L			L			–		–			L	L	L
12	PSP	**E**	**E**	**E**				L		L	L		**E**	**E**	**E**		**E**	**E**
13	TDP-A							L	L	**E**		L	L	L		L	L	L
14	TDP-A	L		L		L		L	L			**E**	L	-	L	L	L	L
15	TDP-A	L		L		–		L	-	L		L	–	–		L	L	
16	TDP-A		L	L		–	L	L				L	–	–		L	L	L
17	TDP-A							L				–	–	–		L		
18	TDP-A	**E**		**E**	**E**	**E**		**E**		**E**	**E**	**E**		**E**	**E**		–	

Apraxic presentations unshaded; PPA presentations shaded

*SNGP* supranuclear gaze palsy, *Eye apra* eye opening apraxia, *Hypomim* hypomimia, *Dysphag* dysphagia, *Hypophon* hypophonia, *Slow tong* slow tongue movements, *Plant extens* extensor plantar response, *Vis inatt* visual inattention, *Stoop pos* stooped posture, *Park gait* parkinsonian gait, **E** early sign, shown in bold, *L* late sign; *–* = no abnormality reported, *empty cells* information not available

### Cognition and behaviour

#### Language

Effortful speech production was present in six patients with tau pathology, often in combination with dysarthria (Table 6). Production in those patients was typically described as slow, although patient 10 with Pick-type pathology was reported to show “slowed initiation, with short rapid bursts of speech”. No patient with TDP pathology showed laboured speech production or articulatory disorder. Their earliest presenting feature was anomia, present both in conversational speech and on picture naming.

**Table 6:** Language, cognition and behaviour

ID	Pathology	Speech			Language			Non-language cognition deficits			Behaviour
		AoS	Dysar	Dyspro	Agram	Sentence comp imp	Anomia	Orient	Spatial	Exec	‘Frontal’ behaviours
1	CBD	**E**	**E**	–	–	–	–	–	–	L	–
2	CBD	**E**	**E**	–	–		–	–	–	**E**	–
3	CBD	**E**	L	–	–	Np	np	np	–	L	–
4	CBD	–	L	–	–	L	–	–	**E** Neglect	L	–
5	CBD	–	**E**	–	–	–	–	–	–	L	–
6	CBD	**E**	–	**E**	–	L	–	–	–	L	L
7	CBD	**E**	**E**	**-**	**E**	**E**	–	–	–	**E**	**E**
8	Pick	–	–	–	–	–	–	–	L	–	–
9	Pick	–	L	–	–	–	L	–	–	L	–
10	Pick	**E**		–	–	L	–	–	–	L	L
11	Pick	–	–	–	(L)	–	L	–	–	–	–
12	PSP		L	**E**	–			**E**	**E** Neglect	**E**	**E**
13	TDP-A	–	–	–	(L)	L	**E**	–	–		–
14	TDP-A	–	–	–	(L)	–	L	–	–	**E**	**E**
15	TDP-A	–	–	–	(L)	L	**E**	–	–	–	–
16	TDP-A	–	–	–	(L)	-	**E**	–	–	–	–
17	TDP-A	–	–	–	–	**E**	**E**	Np	–	L	L
18	TDP-A	–	**E**	–	(L)	L	**E**	**E**	**E**	**E**	**E**

Apraxic presentations unshaded; PPA presentations shaded

*AoS* apraxia of speech (effortful production, articulatory groping or distortion; slowed speech rate, segmentation within words), *Dysar* dysarthria, *dyspros* dysprosody, *agram* agrammatic spontaneous speech, sentence comp imp sentence comprehension impaired, *Orient* orientation in time and place, *Exec* executive functions, **E** abnormality early in course, *L* abnormality late in course, *(L)* likely late but interpretation confounded by severity of anomia, *–* = no abnormality reported, *np* not possible to determine because of communication difficulty, *empty cells* information not available

#### Other cognitive skills

All but two patients were well oriented in time and place and were not clinically amnesic (Table 6). No patient showed problems in visual object perception. Spatial problems were, however, an early feature in three patients. Two patients (4 and 12) showed neglect of the left side of space, elicited by neurological examination and apparent on neuropsychological assessment. Spatial difficulties were confined to patients with an apraxic presentation but did not predict underlying pathology. Difficulties on tests of executive function were demonstrated in most patients at some time during the disease course, impairments being most marked in patients 7, 12, 14 and 18. They showed inattention, poor mental set shifting and response perseverations. Executive impairments were not documented in four patients, although their profound problems in language and gestural communication precluded formal assessment in late-stage disease.

#### Behaviour

Most patients were insightful into their difficulties, and their behaviour was socially appropriate. Nevertheless, some patients exhibited, at presentation or later in the disease course, behavioural changes consistent with frontal lobe dysfunction (Table 6). Recorded features included social disinhibition (patients 7 and 10), emotional blunting and unconcern (patients 7 and 14), loss of sympathy and empathy (patient 12) and altered preference for sweet foods (patients 7 and 12). Neither executive disorder nor ‘frontal’ behavioural change was a predictor of underlying pathology.

### Case histories

Case studies have been published previously of patients 8, 11 and 15. Patient 8, with Pick-type pathology exhibited a circumscribed, symmetrical apraxia [42]. Patient 11, also with Pick-type pathology, presented with a rare, circumscribed disorder of literacy: deep dyslexia and dysgraphia [43]. Apraxia occurred as a late feature. Patient 15, with TDP-A pathology and *GRN* mutation, was profoundly anomic yet he could spell out orally the names of objects that he could not name, suggesting impaired access to the phonological but not orthographic word form [44, 45].

The following are further illustrations of patients, respectively with CBD, Pick’s and TDP-A pathology.

#### Patient 4 (tau-CBD)

This right-handed man was assessed at 69 years complaining of a 3-year history of manual difficulties, affecting daily activities such as writing, using cutlery and dressing. His left hand was more affected than the right. He had also noticed difficulty in reading and calculation but no problems in oral expression or comprehension. He had slowed down generally. On examination, he was insightful and frustrated by his difficulties. The salient abnormality was profound asymmetric apraxia. This was most marked in the left upper limb but also involving orofacial and lower limb movements. Alien limb phenomena were present in the left upper limb, but no myoclonus. Neurological examination also showed increased tone, especially in the left upper limb and exaggerated tendon jerks. There were bilateral grasp reflexes. Neuropsychological testing elicited spatial difficulties, including on tasks with minimal motor demands, such as mental visualisation and spatial matching. There was neglect of the left side of space. Linguistic skills were primarily preserved. His reported problems in reading reflected a difficulty in tracking a line and neglect of the left side of the page. Face and object perception was well preserved. He was well oriented and not clinically amnesic. Executive test performance was compromised by his motor difficulties. A SPECT scan showed hypoperfusion most marked in right temporo-parietal region. He died 2 years after initial assessment aged 71 years. Post-mortem examination revealed bilateral atrophy, particularly affecting posterior parietal regions, and histopathology consistent with CBD.

#### Patient 10 (tau-pick)

This woman presented at the age of 55 years with a 2-year history of progressive difficulties in speech production. She also had difficulty with other voluntary orofacial movements, such as licking her lips, clearing her throat, coughing, and swallowing. She was insightful into her difficulties and had become anxious and despondent. On examination, the dominant feature was a profound orofacial apraxia. She could not smile on request or convey other emotional expressions. She could not maintain eye closure or tongue protrusion. She had difficulty initiating speech, which was produced in short bursts with a stuttering quality. Mild linguistic difficulties were also present: a reduced immediate span, impaired comprehension of complex syntax and errors in spelling. Confrontation naming performance was well preserved. No notable abnormalities were detected in the domains of object perception, face recognition, spatial skills, or memory. Mild difficulties were elicited on an executive test of abstraction and mental set shifting.

Apraxia was largely confined to orofacial movements. She produced limb gestures well, both symbolic and non-representational, the only abnormalities being a tendency to substitute body parts when pantomiming actions and poor persistence when executing alternating hand movements. Neurological examination of the limbs was normal. There was no limb rigidity. An MRI scan showed atrophy with frontal prominence and a SPECT scan bilateral frontotemporal hypoperfusion.

With progression, she could no longer speak and relied on written communication. Words were misspelt and poorly formed with perseverative strokes. She died aged 57 years, 4 years after onset of symptoms. Post-mortem pathological examination revealed severe bilateral damage to motor and premotor regions of the cerebral cortex. The histology was of Pick type, with numerous cells containing tau-positive inclusion bodies (Pick bodies) and frequent swollen, achromatic, tau-positive cells (Pick cells).

#### Patient 17 (TDP-A)

At 62 years, this man developed problems in language expression. He had no physical complaints and at referral, 2 years after onset of symptoms, neurological examination was normal. He lived alone and was independent in all activities of daily living. He continued to drive.

The overriding feature on examination was profound anomia, apparent in conversation and formal testing. He named 0/40 items on an undemanding picture naming test. In conversation, his difficulty retrieving words gave a non-fluent quality, yet pauses were interspersed by fluent, effortless bursts (e.g., “I can’t get the word, flipping heck”). He made no articulatory, phonological, or semantic, errors. Rather, he was unable to generate the word at all. His production difficulties extended to reading, writing, and repeating words. He was typically unable to attempt a response.

In contrast to his preserved manual difficulties in daily life, he exhibited a significant gestural apraxia, which constituted a generalised difficulty in production of symbolic actions, affecting orofacial, upper limb and lower limb movements and affecting right and left limbs equally. Limb actions were inaccurate in terms of internal configuration and position in space, and there was only modest improvement following demonstration. There was a tendency to substitute verbalisations for the required action, e.g., “cough” when asked to produce a cough. Sometimes responses revealed concreteness of thought, e.g., Qu. ‘How would you show you are angry’, Ans. “actually, I’m a person… I never…”; Qu. ‘How would you stub out a cigarette with your foot’, Ans. “I wouldn’t do that”. He performed in the low-normal range on the Brixton spatial anticipation test [46] of executive function. His behaviour was socially appropriate.

Over the following 2 years, there was a gradual diminution in his speech output, which became restricted to stereotyped phrases and ultimately mutism. During the follow-up period, there was no evidence of laboured speech production, articulatory distortion or groping, impaired phonation, or loss of prosody. His problems in verbal communication were mirrored by profound difficulties in gestural communication, both following verbal request and on demonstration: generally, no response was elicited although occasionally he produced an inaccurate response, e.g., he held his hands in front of him when copying a salute. There was a striking disparity between actions in an everyday compared to communicative context. For example, he was unable to attempt to copy the action of drinking from a cup even when the cup was present, yet when offered a cup of tea he spontaneously stirred the tea with a teaspoon and drank from the cup normally. He walked at a normal rate, negotiated the environment without difficulty and seated himself on a chair with ease. At home, he continued to dress and bathe himself unassisted.

His difficulties in language and praxis were interpreted in terms of impaired access to phonological, orthographic, and gestural output systems leading to a generalised disorder of communication. Genetic testing revealed a mutation in the *GRN* gene. He died aged 67 years, 5 years after onset of symptoms. Post-mortem pathological examination revealed severe asymmetric atrophy, most marked in frontal and anterior temporal regions of the left hemisphere, and greater involvement of inferior and middle compared to superior temporal gyri. The histopathology was TDP-type A.

## Discussion

The study highlighted clinical and pathological heterogeneity of apraxia associated with FTLD pathology. Apraxia represented a circumscribed disorder and the dominant presenting symptom in some patients and occurred as a secondary accompaniment to PPA in others. The underlying pathology was most often FTLD-tau but in some cases FTLD-TDP. Tau pathology encompassed not only CBD, but also Pick and PSP subtypes. Such heterogeneity is consistent with the body of evidence that distinct clinical presentations may be associated with CBD pathology [47–49] and conversely, distinct pathologies may underpin a CBS clinical presentation [6, 7, 9]. There were, nevertheless, features of predictive value.

### Apraxia and tau versus TDP pathology

Apraxia presenting as the dominant and relatively circumscribed disorder was a significant predictor of tau pathology. This is in line with previous studies that have emphasised the link between apraxic syndromes and tau [50, 51]. In patients with TDP pathology, apraxia was more likely to occur in the context of PPA and emerge as a secondary or late feature. In patients with tau pathology, the apraxia commonly affected one body part disproportionately, either orofacial or limb movements, whereas dominant apraxia in one body part was not reported in patients with FTLD-TDP pathology.

A notable observation in patients with TDP pathology was relative preservation of activities of daily living and object use in daily life. This suggests that their impaired pantomime of object use constituted one facet of a more general problem in communication, an interpretation in keeping with arguments that pantomime represents a communicative act [52].

### *Apraxia and *subtypes of tau* pathology*

Apraxia as the dominant presenting characteristic was, as expected, most often associated with CBD pathology. Nevertheless, it occurred also in association with Pick type and PSP pathologies. There were some clinical pointers to the form of tau pathology. In patients with CBD pathology, limb apraxia was typically markedly asymmetric and associated with an asymmetrical predominance of atrophy/hypoperfusion found on neuroimaging. By contrast, the limb apraxia was bilateral and relatively symmetrical in most patients with Pick-type pathology, and, apart from patient 11 who presented with a language disorder, atrophy was symmetrical. Limb rigidity was reported as an early feature in all patients with CBD pathology but was typically a late development in other patients including those with Pick pathology. These distinctions are relevant with respect to current diagnostic criteria for CBD [1]. A common clinical presentation of CBD, recognised by those criteria, is CBS. A diagnosis of “probable CBS” is based on an asymmetric presentation of limb rigidity, dystonia, and/or myoclonus, together with orobuccal or limb apraxia, corticosensory deficit and/or alien limb phenomena. In this study, patients with CBD pathology typically met criteria for “probable CBS” early in the disease course, whereas patients with Pick-type pathology did not. Those with Pick-type pathology fulfilled criteria only for “possible CBS”, by virtue of the symmetrical presentation, and, given the late emergence of limb rigidity, only late in the disease course. The findings highlight the need for caution in assuming CBD pathology in patients with symmetrical apraxia in the absence of appendicular rigidity. It is worthy of note that patients 8 and 10 with Pick-type pathology had been suspected of having a progressive apraxic syndrome distinct from CBD at the time of their initial referral. Patient 8, like other cases [42, 53, 54], had been classified as having a slowly progressive apraxia.

The single patient with PSP pathology exhibited at presentation an asymmetric limb apraxia consistent with CBS. He also showed early supranuclear gaze palsy, hypomimia, parkinsonian gait and falls, consistent with PSP syndrome. Yet, PSP signs were also documented in some patients with CBD pathology, so did not have diagnostic specificity.

### Apraxia of speech

In all patients in the current series, including those with FTLD-TDP pathology, apraxia involved orofacial movements at some time during the disease course. Difficulties included facial gestures and action pantomimes and included production of speech sounds as well as non-verbal oral movements. Should then they be considered to exhibit apraxia of speech (AOS)?

AOS is defined as a motor speech disorder that affects the planning and programming of movements required for speech production [50, 55–57]. It has typically been linked to tau pathology, particularly CBD and PSP [51]. The precise cognitive underpinning of AOS has been controversial [56, 58]. Nevertheless, there are recognised clinical characteristics: articulatory distortions and groping, distorted sound substitutions (phonetic type), or else slowed speech rate with segmentation within multisyllabic words (prosodic type) [51]. All patients with tau pathology in whom orofacial apraxia was the dominant presenting symptom demonstrated those characteristics of AOS. By contrast, no patient with TDP-A pathology exhibited those articulatory and prosodic hallmarks of AOS suggesting that their difficulties with speech production lay at a level that was not primarily motoric. The difficulties were interpreted as occurring at a higher order linguistic level. As illustrated in the case history of patient 17, patients typically produced an utterance correctly or else simply could not attempt a response at all. Patient 17’s difficulty, like that of patient 15 published previously [44, 45] was attributed to impaired access to output pathways for communication: phonology, orthography, and gesture. That is, the problems in praxis mirrored those in other language domains and constituted one dimension of a generalised loss of communication output systems. It is worth noting in this regard that patients with TDP-A pathology were not reported to compensate for their spoken communication difficulties by use of limb gesture, which might otherwise be expected in patients with prominent anomia. On the other hand, patient 17, as well as other patients with TDP-A and *GRN* mutations, were recorded as showing relative preservation of praxic skills in relation to activities of daily living, contrasting with their severe apraxia on formal testing. These features support the notion that patients’ apraxia reflects their widespread disorder. of communication. The purported absence of AOS in these patients is in keeping with other reports [59, 60] of a lack of association between AOS and *GRN* mutations. It is noteworthy, however, that a recent case report [61] described AOS as an atypical presenting characteristic in a patient with a progranulin mutation and TDP-A pathology, suggesting that AOS cannot be definitively excluded.

### Anomia and G*RN mutations*

The findings have implications for understanding the language disorder associated with *GRN* mutations. Anomia, as found in the present patients, is a common presenting feature of *GRN*-related PPA [62, 63], and frequently classified as logopenic variant (lv) PPA [64–66]. Nevertheless, it has been observed that *GRN*-associated PPA may not be entirely consistent with lvPPA, suggesting a distinct phenotype [64]. The study of patient 15 [44, 45] indicated that, at referral, he could spell out orally the names of objects (e.g., c-a-t) that he could not name (e.g., cat), indicating a dissociation between access to an object name’s phonological form and to its orthography. His naming difficulty could not be interpreted simply as one of word finding. Similarly, the naming problem in patient 17 was ascribed to impaired phonological and orthographic output systems. The *GRN*-related anomia of patients in the present series likely has a linguistic substrate that differs from that of conventional lvPPA.

### CBS clinical syndrome and GRN mutations

Patient 18 is distinct from others with TDP-A pathology in which he presented with a CBS-type clinical syndrome. The severe apraxia, which affected all four limbs, most marked on the left, combined with asymmetric extrapyramidal signs lead to an initial presumptive clinical diagnosis of CBD. His presentation reinforces earlier findings of an association between a CBS clinical presentation and *GRN* mutations [7, 67–71]. The patient’s condition was severe at the time of initial assessment, complicating interpretation of cognitive underpinnings. Nevertheless, there are features of note. Many of his apraxic ‘errors’ constituted a total absence of response rather than faulty execution. Similarly, he generated no spontaneous speech, nor could he recite on request verbal series such as the months of the year. His recitation of the months was facilitated by phonological cueing, confirming that he had no articulatory difficulty, and raising the possibility of impaired phonological access. The patient was the brother of patient 17, described in the case report and, uniquely amongst the TDP-A group, he had predominantly right rather than left-sided atrophy. Moreover, post-mortem examination revealed severe posterior parietal lobe atrophy. It might be speculated that he shared with his brother impairments in access to phonological, orthographic and gestural output systems, the prominence of apraxia, as opposed to aphasia, reflecting both the prominence of parietal lobe involvement and right-sided distribution of atrophy.

Patient 18 exhibited more prominent ‘frontal’ behavioural features than typically seen in CBD: concreteness, verbal and motor perseverations, inattention and poor persistence, and a history of food cramming. This observation is consistent with neuroimaging findings that although CBS is associated with grey matter loss in premotor cortices, supplementary motor areas and insula regardless of FTLD pathology, there is greater involvement of prefrontal cortex associated with TDP compared to CBD and PSP pathology [72].

Most patients in the present cohort had no relevant family history. Familial cases were strongly associated with mutations in the *GRN* gene, suggesting that genetic screening for *GRN* should be considered in CBS patients with a positive family history.

### Alien limb

Alien limb phenomena are a common accompaniment to CBS [1, 73]. The characteristics are variable [73], consistent with the notion that alien limb is not a uniform entity and may involve distinct subtypes [74, 75]. In the present series, alien limb phenomena were recorded exclusively in association with an apraxic clinical presentation. Their presence did not, however, reliably predict underlying pathology since they occurred in patients with CBD, Pick’s and TDP-A pathology. Comparisons between those pathologies are limited by the small number of patients. It may be relevant though that involuntary levitation and wandering of the limb in space and mirroring of movements by the opposite hand occurred only in association with tau pathologies, that striking inter-manual conflict and tonic grasping characterised the patient with Pick pathology, and both tonic grasping and involuntary release of objects were present in the patient with TDP-A pathology.

### Cognitive and behavioural characteristics

Executive difficulties and alterations in behaviour were documented in a minority of patients early in the course of disease and in others with disease progression. Such findings underscore the overlap between CBS and PPA syndromes and behavioural variant FTD [1, 6, 76].

### Limitations

The study cohort was small, necessitating caution in generalising to the population of FTLD. This is particularly so given that statistical tests were uncorrected for multiple comparisons. Patients differed in terms of the severity of illness at referral. Patient 1 was in the later stages of illness, so that the evolution of symptoms could only be inferred from informant reports and earlier medical correspondence. Patient 18, although having shown symptoms for only 3 years, was profoundly affected at the time he was first assessed, again complicating the interpretation of symptom evolution. Imaging was limited to routine clinical structural and/or functional scans, sometimes carried out in a different hospital, and not always synchronous with their clinical assessment. For some, only scan reports were available. Hence, neuroimaging correlations and potentially informative comparisons, such as the degree of involvement of parietal regions and supplementary motor area, were not viable. It is worthy of note, however, that parietal atrophy and/or hypoperfusion was present to some degree in all patients in keeping with its established role in apraxia.

AD co-pathology was reported in three patients. This was, however, of minor severity, so unlikely to contribute significantly to the clinical picture. No patient with tau-Pick or TDP-A pathology had additional AD pathology.

The retrospective nature of the study creates challenges in interpretation of data: whether absence of report of a symptom/sign equates with absence of that symptom or constitutes missing data. Nevertheless, patients included in the study were all assessed in a single specialist neurological centre by the same neurologists and neuropsychologists and using the same methodology, as previously described [33], and a uniform reporting style. It was considered unlikely that group differences arose spuriously. Importantly, the clinical documentation of apraxia in patients’ records was independent of knowledge of underlying pathology.

The study focussed only on that subset of patients with FTLD pathology in whom apraxia had been highlighted as a prominent feature, at presentation or during the disease course. As such, it encompassed principally patients with a CBS-like clinical syndrome. The study did not address the full range of potential performance breakdown on tests of apraxia, for example, sequencing problems in PSP syndrome [28] and problems in object use as found in semantic dementia [27].

## Conclusion

The study confirms that apraxia is associated with a range of subtypes of FTLD pathology and is not exclusive to tau-CBD. Distinct apraxia characteristics appear predictive of pathological subtype. Not all FTLD patients with significant apraxia meet criteria for CBS. Moreover, the classification of CBS, even when applicable, may potentially mask different mechanisms underpinning the apraxia. Notably, in patients with TDP-A pathology, the apraxia may be one manifestation of patients’ generalised communication disorder. There is a need for studies to address prospectively hypothesised distinctions between phenotypic variants of apraxia associated with FTLD.

## Data Availability

Coded data are presented in the Tables. Patients’ clinic files, from which data are drawn, are not available to protect patient confidentiality. For further information, contact the corresponding author.
